# Host-cell interactions in HBV infection and pathogenesis: the emerging role of m6A modification

**DOI:** 10.1080/22221751.2021.2006580

**Published:** 2021-12-01

**Authors:** Anastasiya Kostyusheva, Sergey Brezgin, Dieter Glebe, Dmitry Kostyushev, Vladimir Chulanov

**Affiliations:** a National Medical Research Center of Tuberculosis and Infectious Diseases, Ministry of Health, Moscow, Russia; b Scientific Center for Genetics and Life Sciences, Division of Biotechnology, Sirius University of Science and Technology, Sochi, Russia; c National Reference Center for Hepatitis B Viruses and Hepatitis D Viruses, Institute of Medical Virology, Justus Liebig University of Giessen, Giessen, Germany; d Department of Infectious Diseases, Sechenov University, Moscow, Russia

**Keywords:** Epitranscriptomics, hypoxia, liver cancer, interferon, METTL, ALKBH, HCC

## Abstract

Hepatitis B virus (HBV) is a DNA virus with a complex life cycle that includes a reverse transcription step. HBV is poorly sensed by the immune system and frequently establishes persistent infection that can cause chronic infection, the leading cause of liver cancer and cirrhosis worldwide. Recent mounting evidence has indicated the growing importance of RNA methylation (m6A modification) in viral replication, immune escape, and carcinogenesis. The value of m6A RNA modification for the prediction and clinical management of chronic HBV infection remains to be assessed. However, a number of studies indicate the important role of m6A-marked transcripts and factors of m6A machinery in managing HBV-related pathologies. In this review, we discuss the fundamental and potential clinical impact of m6A modifications on HBV infection and pathogenesis, as well as highlight the important molecular techniques and tools that can be used for studying RNA m6A methylome.

## Introduction

**Figure 1.**
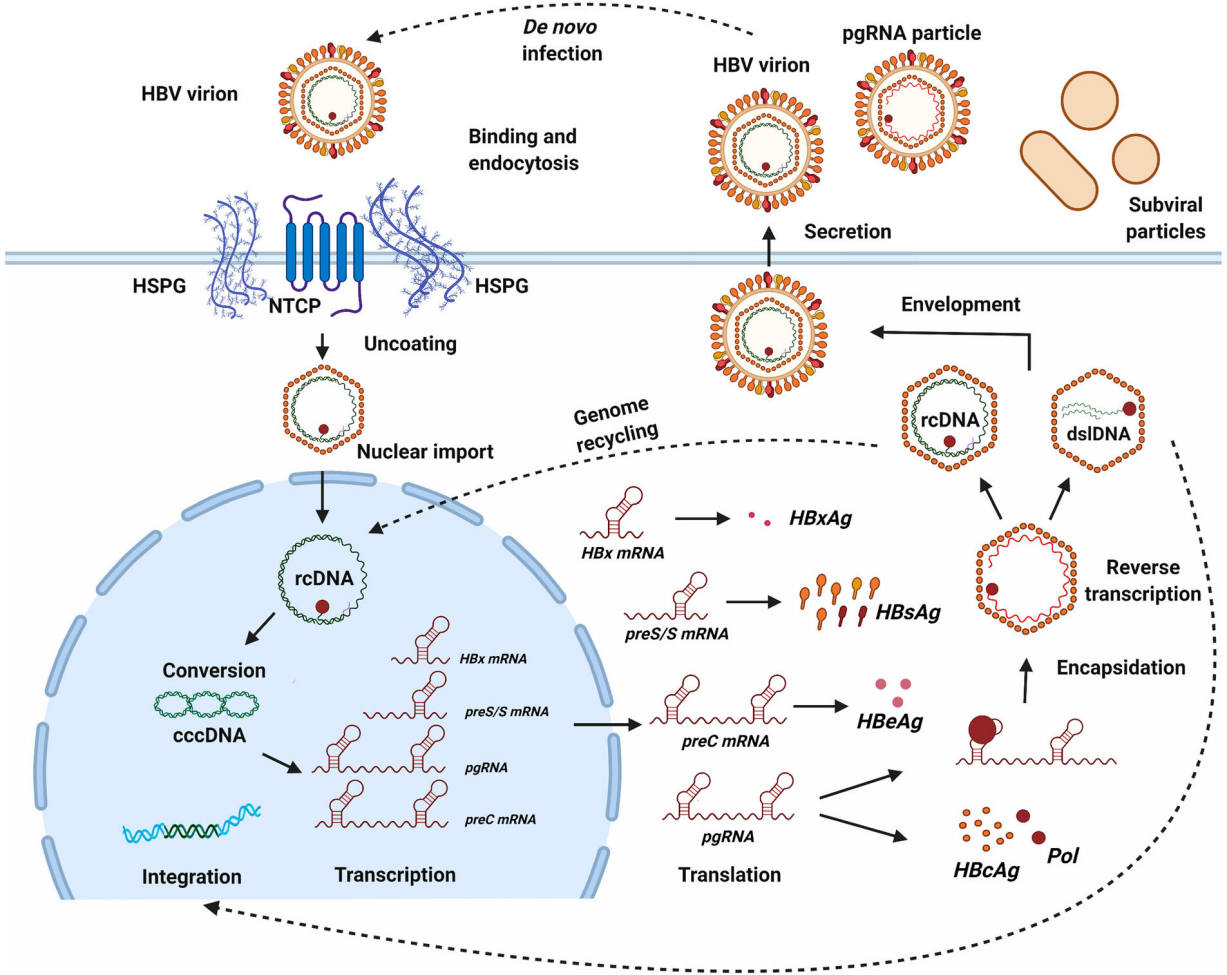
HBV life cycle. HBV enters hepatocytes through the initial attachment to heparan sulfate proteoglycans and then by highly-specific interaction with sodium taurocholate cotransporting polypeptide (NTCP) receptor followed by virion uncoating and transport to the nucleus. In the nucleus, rcDNA is converted into cccDNA by a multi-step process with the assistance of host factors. cccDNA is a template for transcription of all viral RNAs (preC RNA, pgRNA, preS RNA, X RNA and additional spliced isoforms). All viral RNAs contain 3′-end ϵ stem loop, while pgRNA and preC mRNA also harbour 5′-end ϵ stem loop. HBV RNAs are exported in the cytoplasm for protein translation. pgRNA is then selectively packaged into newly formed nucleocapsids, where it is reverse transcribed to produce rcDNA. Nucleocapsids can be enveloped to form a mature virion and released from cells or transported to the nucleus to replenish cccDNA pool. If reverse transcription is interrupted, virions containing pgRNA are released from cells. Double-stranded linear HBV DNA (dslDNA), produced due to aberrant reverse transcription, serves as the major source of viral integration. HBV DNA integrations represent a replicative dead-end for the virus, but serves as a template for viral RNA and protein production [1].

Hepatitis B virus (HBV) is a hepatotropic virus that causes acute and chronic hepatitis B (CHB), one of the most widespread infectious diseases in the world. CHB often progresses to cirrhosis and hepatocellular carcinoma (HCC). HBV life cycle is depicted in Figure 1. HBV has a partially double-stranded DNA (rcDNA) genome containing four overlapping open reading frames encoding viral proteins HBs, HBc, HBe, HBx, and Polymerase (Pol). After entering the cell, viral nucleocapsids are transported to the nucleus, where rcDNA is converted into covalently closed circular DNA (cccDNA).

The intranuclear episomal cccDNA pool is the template for all viral RNAs that carry a 5′-cap and a 3′-polyA tail which are identical to cellular messenger mRNAs. After transport into the cytoplasm the pre-genomic RNA (pgRNA) is packaged into newly formed capsids, and reverse-transcribed to rcDNA by the viral polymerase (reviewed in [1]).

Dozens of different post-transcriptional modifications of RNA have been described. N^6^-methyladenosine (m^6^A) is the most common post-transcriptional modification, found on eukaryotic RNA [2]. M^6^A modification affects critical aspects of RNA function and cellular physiology, and has also been found on viral RNA, including HBV RNA. The role and mechanisms of m6A modification in cell function and viral replication were reviewed elsewhere [3]. Regulation of m6A modification is a reversible process. M6A methyltransferases (**writers**) METTL3, METTL14, and Wilms tumour associated protein (WTAP) co-transcriptionally add m6A modifications in the nucleus, while m6A **erasers** fat mass and obesity-associated protein (FTO) and AlkB homolog 5 (ALKBH5) remove these marks. Several groups of m6A **readers** like a group of 5 YT521-B homology (YTH) domain-containing proteins YTHDC1-2 and YTHDF1-3, and additional readers recognize m6A-methylated RNA and contribute to their function and processing. In the m6A methyltransferase complex, METTL3 exhibits catalytic methyltransferase activity and mediates m6A addition to RNA in the nucleus, METTL14 is an RNA binding platform that supports the integrity of the METTL3-METTL14 complex and strengthens catalytic activity of METTL3 [4]. WTAP interacts with the METTL3-METTL14 heterodimer, driving the localization of the complex to the sites of pre-mRNA splicing, and promotes methyltransferase activity [5]. Other accessory proteins in the complex (VIRMA, ZC3H13, etc.) regulate the stability, cellular localization, and m6A methylation of RNA 3′-UTR. M6A writers are mainly nuclear but can also be found in or shuttled to the cytoplasm. Demethylases FTO and ALKBH5 can demethylate m6A-modified RNA, deleting the epigenetic mark and thus establishing the reversibility of m6A modification. ALKBH5 protein is predominantly nuclear and scantly localized in the cytoplasm [6]. FTO protein resides both in the nucleus and the cytoplasm, generating a mobile fraction that shuttles between the two cellular compartments [7]. The effects of m6A modification on RNA depend on the recognition of m6A-modified RNA by different m6A binding proteins. YTHDC1-2 and YTHDF1-3 proteins bind RNA in an m6A-dependent manner irrespective of RNA length [8]. YTHDF1 mediates mRNA translation [9], whereas interaction of m6A-marked RNA with YTHDF2 reduces RNA stability and promotes mRNA degradation [10]. Binding of other readers to m6A RNA occurs indirectly and possibly requires additional co-factors to bind RNA [11]. Myriads of cellular proteins may act as readers of m6A marks, permitting widespread regulatory control over transcriptomics [12].

In recent years, understanding the role of m6A in regulating RNA and cellular processes has improved. In this review, we discuss the interplay between HBV infection and m6A methylation of both viral and cellular RNA; and the possible implication of m6A-marked RNA in the development of HBV-induced HCC.

## Interaction between HBV life cycle and M6A methylation

M6A methylation was detected in viral RNA upon infection by numerous viruses. Recent studies demonstrate that m6A factors regulate the viral life cycle and may profoundly affect outcomes of virus-host interactions. Effects of m6A modification can regulate viral replication directly (by adding m6A to genomic RNA and/or mRNA of viruses) or indirectly (by altering expression of genes or fate of corresponding RNA involved in viral replication).

**Figure 2.**
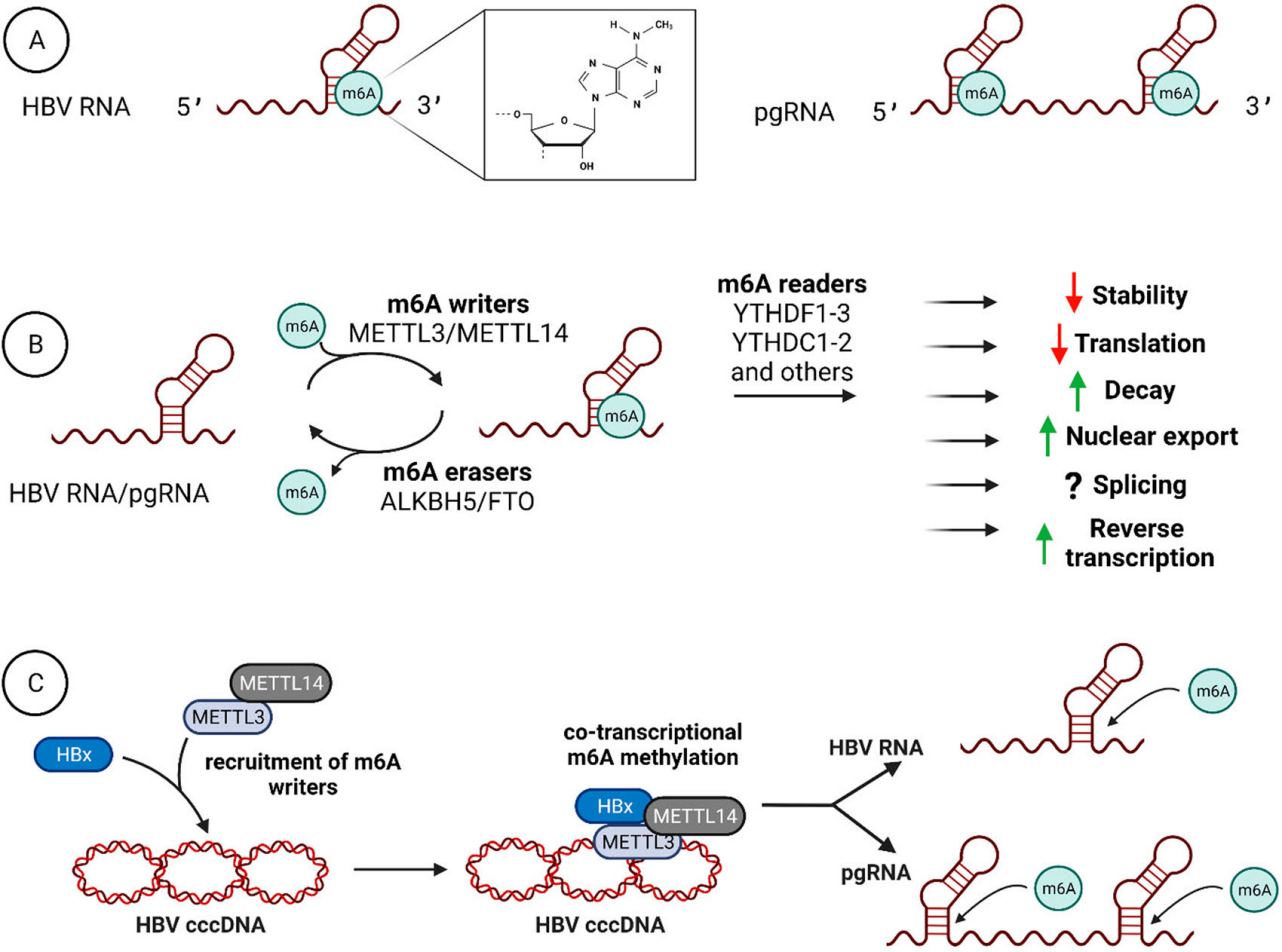
m6A modification of HBV RNA and its functional effects. (A) HBV RNA is methylated only at a single position, in the typical DRACH motif (D = A, G, or U; R = A or G; and H = A, C, or U), at A1907 at the 3′ end. As pgRNA contains two copies of the DRACH motif due to terminal redundancy, it is m6A methylated both at 3′ and 5′ ends. (B) HBV RNA/pgRNA is m6A modified by m6A writers (METTL3/METTL14) where METTL3 uses S-adenosyl methionine as a methyl donor, and METTL14 functions as an RNA-binding platform. M6A-modified HBV RNA/pgRNA is then recognized by specific reader proteins, such as YTHDC1-2, YTHDF1-3, IGF2BP1-3, eIF3, FMRP, FXR1, FXR2, SND1, hnRNPA2B1, hnRNPC, hnRNPG, etc. The effects of m6A modification typically depend on the recognition by different m6A readers. Interaction with YTHDF2 and YTHDF3 reduces stability and accelerates the decay of m6A-modified HBV RNA and reduces HBV protein production. YTHDC1 and FMRP readers promote export of HBV RNA from the nucleus into the cytoplasm and affect HBV pgRNA encapsidation process. M6A modification at the 5′ end of HBV pgRNA promotes reverse transcription. (C) HBx induces nuclear import of METTL3/METTL14 and recruits the m6A writers to the HBV cccDNA, inducing co-transcriptional m6A modification of HBV RNA/pgRNA.

Imam et al. (2018) showed that m6A methylation of HBV RNA regulates the HBV life cycle [13]. HBV RNA isolated from cell lines in vitro and from the liver of CHB patients is m6A-methylated (Figure 2(A)). Knocking down METTL3/METTL14 markedly reduces the levels of m6A in HBV RNA and increases their stability and expression of HBsAg and HBcAg; conversely, depleting FTO/ALKBH5 reduces HBV protein production (Figure 2(B)). These data suggest that the presence of m6A on HBV RNA accelerates HBV RNA degradation, reducing RNA stability and production of viral proteins. At the same time, Imam et al. discovered a reduction in HBV DNA in METTL3/METTL14 knockdown cells, indicating that m6A increases reverse transcription of pgRNA and formation of HBV DNA (Figure 2(B)). This duality in the effects of m6A on pgRNA and total RNA can be explained by the presence of m6A both 3′ and 5′ ends of pgRNA due to terminal redundancy, whereas in the rest of HBV RNA, m6A is present only at the 3′ end (Figure 2(A)). Thus, methylation at both ends in pgRNA promotes reverse transcription of pgRNA and increases the levels of core-associated HBV DNA, whereas methylation at just the 3′ end of other viral RNA facilitates degradation of HBV transcripts, reducing protein synthesis.

The exact sites of m6A modifications in HBV RNA and pgRNA were identified at positions 1905–1909, in which A1907 nucleotide was m6A-methylated. This site is conserved among all HBV genotypes [13]. The viral potential G-quadruplex (PGQ)-forming sequences may provide the structural framework for installing m6A modifications. HBV m6A sites on pgRNA are located near two PGQ that can fold to G-quadruplexes, support m6A modification, and regulate viral processes [14]. The role of m6A modifications at 3′ and 5′ ends of pgRNA was directly assessed by generating single-nucleotide mutants with disrupted m6A site. Mutating the 3′ m6A motif revealed that m6A modification of this terminus decreases RNA stability and production of viral proteins. Accelerated degradation of 3′ methylated HBV RNA is likely mediated by interaction with reader YTHDF proteins, as depleting YTHDF2 (the major destabilizer of m6A-RNA) and YTHDF3 (which facilitates interaction between YTHDF2 and m6A-RNA) increases HBV protein levels. Therefore, m6A-modified HBV RNA is likely as stable as unmodified RNA if YTHDF proteins are depleted. In contrast, adding m6A at the 5′ stem positively affects reverse transcription by mechanisms that are not yet completely understood [13].

In a different study, using a targeted CRISPR RNA demethylation system (TRADES) generated based on CRISPR/Cas fused to m6A demethylases (ALKBH5 or FTO) it was possible to erase m6A marks at specified loci [15]. Targeting HBV RNA by TRADES resulted in a ∼2-fold increase in HBV cccDNA copy numbers. It remains unclear how HBV cccDNA is more actively replenished upon m6A depletion. Deleting m6A from pgRNA by TRADES should limit reverse transcription and reduce HBV DNA levels. Less HBV DNA should result in less active replication, less effective re-import of HBV rcDNA back to the nucleus, and less efficient HBV cccDNA replenishment [16]. These contradictory results may be related to the large quantities of ALKBH5/FTO delivered intracellularly by TRADES, which may contribute to the expansion of the cccDNA pool. Another explanation is that removing m6A from 3′ ends of HBV RNA increases HBV protein levels, including the transactivator HBx, which can contribute to more active HBV replication [17]. To conclude, engineering m6A in HBV infection is a fascinating new area of research that is now accessible due to the development of CRISPR-based tools. Dissecting the role of m6A marks established on different transcripts and bound by different readers and co-factors upon HBV replication can bring new insights into the HBV life cycle and potentially into the concept of therapeutic control over HBV transcription and cccDNA silencing.

HBV transactivating protein HBx, which plays a primary role in HBV transcription, can also mediate m6A modifications of viral transcripts (Figure 2(C)). HBx-defective HBV reduces m6A modification of HBV transcripts [18]. The authors showed that HBx, METTL3, and METTL14 proteins were all bound to cccDNA. Thus, HBx may contribute to co-transcriptional m6A modification of viral RNA. Although HBx was shown to interact with METTL3-METTL14 complex in nuclear and cytoplasmic fractions, only nuclear HBx could induce m6A methylation of viral RNA. HBx protein also induces nuclear import of METTL3/METTL14 complex during HBV replication. In the absence of HBx, m6A modification of viral RNA does not occur. Thus, HBx, as the major regulator of HBV transcription, induces the import of two major m6A writers into the nuclei of HBV-infected cells and serves as a co-factor for the binding of the METTL3/METTL14 complex to cccDNA to co-transcriptionally add m6A marks on HBV transcripts. As pgRNA is accessible to cellular factors mainly in the nucleus, HBx-induced nuclear import of METTL3/METTL4 to establish m6A modification of pgRNA appears virologically prudent for supporting HBV persistence.

Another parameter of HBV RNA influenced by m6A is its cellular distribution. Reducing levels of m6A on HBV RNA by using HBV genomes with mutated m6A sites or by knocking down METTL3/METTL14 results in preferential accumulation of HBV RNA in the nucleus [19]. This suggests that during HBV infection, HBV RNA carries m6A modifications that promote their export from the nucleus into the cytoplasm. Two factors responsible for the transport of m6A-modified HBV transcripts are FMRP and YTHDC1 readers. Depleting these factors leads to accumulation of HBV RNA in the nucleus and reduction of core-associated pgRNA, HBV rcDNA, and overall cccDNA levels. Thus, FMRP and YTHDC1 might affect HBV pgRNA encapsidation process by regulating RNA nuclear transport.

## M6A Methylation in (Dys)regulation of innate immunity

In recent years, evidence has emerged regarding the role of m6A in regulating immunity and antiviral responses [20]. In particular, m6A modifications were shown to define the strength and longevity of innate immune response activation.

Type I interferon (IFN) response is intricately regulated, balancing between augmenting and suppressing signals to ensure effective antiviral response [21]. Winkler et al. reported that m6A modification of IFN RNA can serve as one mechanism to regulate IFN signalling [20]. Indeed, IFNβ mRNA can be m6A-modified, reducing mRNA stability and restricting the duration of IFN type I immune response. However, m6A modification can both positively and negatively impact antiviral innate immune response. Several studies revealed that m6A machinery acts as a negative regulator of antiviral immune responses. DDX46 was shown to interact with mRNA of antiviral factors *Mavs*, *Traf3*, and *Traf6*, and recruit to them the m6A eraser ALKBH5. M6A demethylation of these RNAs results in retention of *Mavs*, *Traf3*, and *Traf6* in the nucleus, thus restricting IFN type I response [22]. Suppression of antiviral responses was also shown for YTHDF3 reader protein, which negatively regulates ISG expression by binding to mRNA of transcriptional co-repressor FOXO3 and promoting its translation [23]. In contrast, a study using an HSV-1 infection model showed that m6A can promote antiviral immunity [24]. The nuclear DNA sensor hnRNPA2B1 forms a complex with viral DNA and translocates to the cytoplasm, inducing IFN-α/β response. hnRNPA2B1 recruits FTO demethylase and prevents its interaction with RNA of antiviral factors CGAS, IFI16, and STING, thus activating IFN-α/β and amplifying innate immune responses.

The hallmark of antiviral immune response is the ability to detect viral nucleic acids among the host cell’s transcriptome (i.e. distinguishing self vs non-self) and trigger the IFN response and production of pro-inflammatory cytokines. As outlined previously [25], chemical modifications of nucleic acids, including m6A, prevent recognition of endogenous RNA by nucleic acid sensors and thus control antiviral responses. As such, m6A serves as a molecular marker to distinguish self from non-self RNA.

Indeed, incorporation of m6A into RNA was shown to inhibit TLR3/7/8 activity [26], as well as abolish recognition by RIG-I [27]. However, viruses have evolved to exploit these mechanisms to evade immunity and maintain persistent infection. HCV RNA and HIV are weakly recognized by RIG-I when m6A-modified [28,29]. In line with this, human metapneumovirus devoid of m6A modifications is more efficiently sensed by RIG-I, leading to higher IFN expression levels [30]. During vesicular stomatitis virus infection, METTL3 translocates to the cytoplasm, methylating viral RNA to trick immune surveillance mechanisms. This reshapes viral RNA structure and makes it less sensitive to cytoplasmic RNA sensors [31]. Similar phenomena are observed during SARS-CoV-2 infection [32].

**Figure 3.**
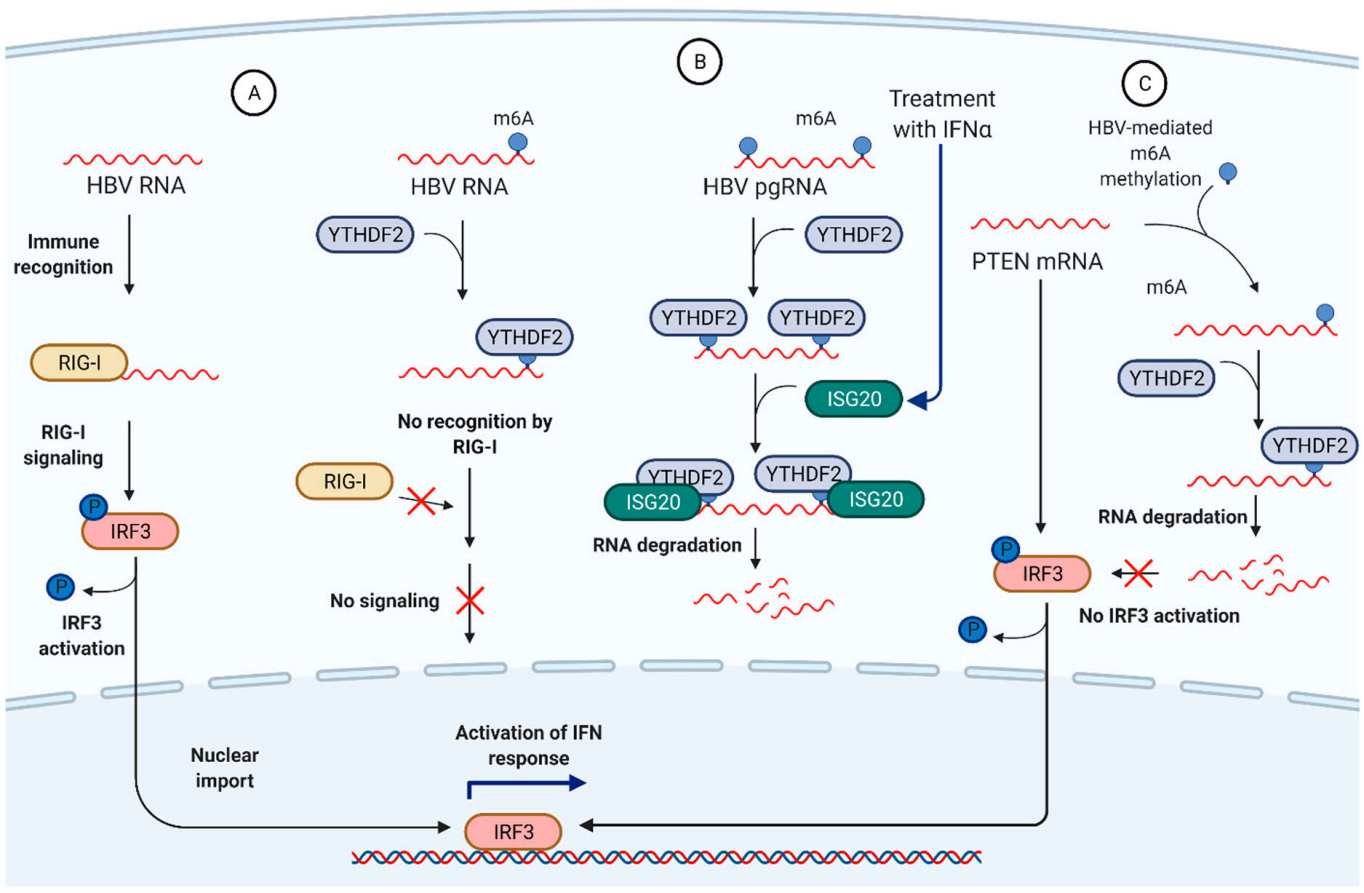
Interplay between innate immune responses and m6A-modified HBV RNA. (A) HBV RNA devoid of m6A marks is readily recognized by cytoplasmic RNA sensor RIG-I followed by activation of the downstream RIG-I signalling, dephosphorylation of IRF-3 and activation of anti-viral IFN responses which contribute to HBV RNA degradation. In contrast, m6A-modified HBV RNA is coupled with YTHDF2 which shields viral RNA from recognition by RIG-I. While RIG-I was found to recognize 5′-ϵ region of HBV pgRNA inducing type III IFN response and preventing interaction of pgRNA with viral Polymerase [33], some evidence suggests that RIG-I is unable to recognize HBV RNA [34,35]. Additionally, HBV can efficiently suppress or bypass RIG-I recognition [36,37]. (B) Treatment of HBV-infected cells with IFNα activates expression of interferon-stimulated genes, including an RNA exonuclease molecule ISG20. In turn, ISG20 can interact only with m6A-modified and YTHDF2-bound HBV RNA, resulting in rapid RNA degradation. (C) In uninfected cells, PTEN mRNA can activate IRF-3 and induce IFN signalling, thus contributing to antiviral immune responses. Upon HBV infection, HBV HBx protein mediates recruitment of METTL3/METTL14 complex to PTEN mRNA and its m6A methylation. Methylated PTEN mRNA is then bound by YTHDF2 reader protein, reducing its stability and promoting PTEN mRNA decay.

HBV is considered a “stealth” virus that largely avoids detection by infected hepatocytes [35,38]. This stealth mode is mediated by the lack of IFN signalling and expression of ISGs in infected cells [39]. However, recent reports challenged the stealth nature of HBV by demonstrating that HBV actively counteracts and subverts antiviral responses [40,41]. At the same time, HBV RNA m6A modification or re-shaping of the m6A methylome of host cells as another level of immune escape has recently come to light. HBV has long been known to suppress or bypass RIG-I/MAVS signalling [36,37]. Kim et al. showed that m6A-deficient HBV RNA exhibits enhanced RIG-I sensing, stimulating IRF-3 activation and IFN signalling (Figure 3(A)) [28]. Silencing METTL3/METTL14 increased IRF3 phosphorylation in HBV-infected cells, whereas their overexpression had the opposite effect. Similar to other viruses, m6A modification of HBV RNA disrupts its recognition by RIG-I sensor. HBV pgRNA harbouring a mutation at the 5′-end, abolishing m6A modification, led to a remarkable increase in RIG-I recognition of viral RNA [28]. The underlying mechanism is related to the function of the reader protein YTHDF2, which competes with RIG-I for binding the m6A site of HBV RNA. Depleting YTHDF2 increases the interaction of HBV RNA with RIG-I. Notably, both suppression of RIG-I/MAVS [42] and m6A modification of HBV RNA [18] are mediated by HBx protein, suggesting another intriguing role of this multifunctional viral regulator in the immune evasion processes.

A remarkable example of the opportunity to harness m6A for developing antiviral approaches is the role of m6A in IFN treatment of HBV. IFN treatment suppresses HBV replication and is a common option to treat chronic infection [43]. ISG20 induced by IFN-α treatment interacts with the epsilon stem-loop of HBV pgRNA, which harbours an m6A site and degrades m6A-modified pgRNA (Figure 3(B)) [44]. Disrupting the m6A site makes HBV pgRNA resistant to ISG20. Moreover, anti-HBV activity of ISG20 is mediated by direct interaction of ISG20 with YTHDF2. YTHDF2 enhances antiviral activity of ISG20 by recruiting it to the m6A site of HBV pgRNA [44].

Another way to influence the innate immune response is by modulating m6A levels of antiviral transcripts. HBV can inhibit induction of IFN signalling by increasing m6A modification of PTEN mRNA, a tumour suppressor factor that is also involved in regulating antiviral immune responses (Figure 3(C)). PTEN induces dephosphorylation of IRF-3 at Ser-97, resulting in IRF-3 nuclear import and IFN synthesis [45]. HBV enhances m6A modification of PTEN mRNA, contributing to its instability and decline of protein levels. This suggests that HBV-mediated PTEN mRNA degradation negatively influences IFN synthesis. HBV-induced elimination of PTEN is now proposed as an important mechanism by which HBV evades host immunity [46]. So far, it is the only example of HBV-induced host methylome manipulation to disrupt immune surveillance mechanisms. Further analysis of the m6A methylome in patients with chronic HBV infection and on different therapeutic regimens may reveal molecular networks that underlie efficacy and failures of antiviral treatments and illuminate novel targets for drug development.

## M6A and hypoxia: the potential link to HCC development

The liver is an organ with varying levels of partial oxygen pressure, showing low (4–8%) O_2_ concentrations in different metabolic zones [47]. A recent study by Wing et al. [48] identified that hypoxia stimulates HBV transcription and HBV DNA secretion via HIF. Investigating m6A modifications induced by HBV in hypoxic conditions is of particular interest, as hypoxia is known to systematically reprogramme the m6A epitranscriptome in cells. HBV is tightly intertwined with hypoxia and m6A modifications. HBx-interacting protein (HBXIP), an oncoprotein that interacts with the C-terminus of HBx, elevates METTL3 levels and accelerates tumour progression. Overexpression of HBXIP mediates m6A modification of HIF-1α mRNA. These conditions provoke cell proliferation, migration, metastasis, and reprogramming of cell metabolism [49].

Tumour metastasis and cancer progression are promoted in hypoxic conditions by HIF-1α, which activates transcriptional networks. HIF-1α is highly overexpressed in HCC. Upon hypoxia, HIF-1α enters the nucleus, forms a heterodimer with HIF-1β, and activates target genes [50]. Hypoxia also contributes to HCC development by m6A-related mechanisms. HIF1 activates transcription of downstream target genes in response to hypoxic stress [51], including YTHDF1. YTHDF1, in turn, induces translation of ATG2A and ATG14 (factors associated with HCC malignancy) by binding to corresponding mRNAs [52]. Increased YTHDF1 expression correlates with poor prognosis in HCC patients. Targeting YTHDF1 may thus be a promising strategy for treating HCC. The opposite role is likely attributed to YTHDF2, which can inhibit proliferation and growth of liver cancer by destabilizing EGFR. In contrast to YTHDF1, hypoxia suppresses YTHDF2 levels.

## Could M6A be a driver or predictor of liver carcinogenesis?

**Figure 4.**
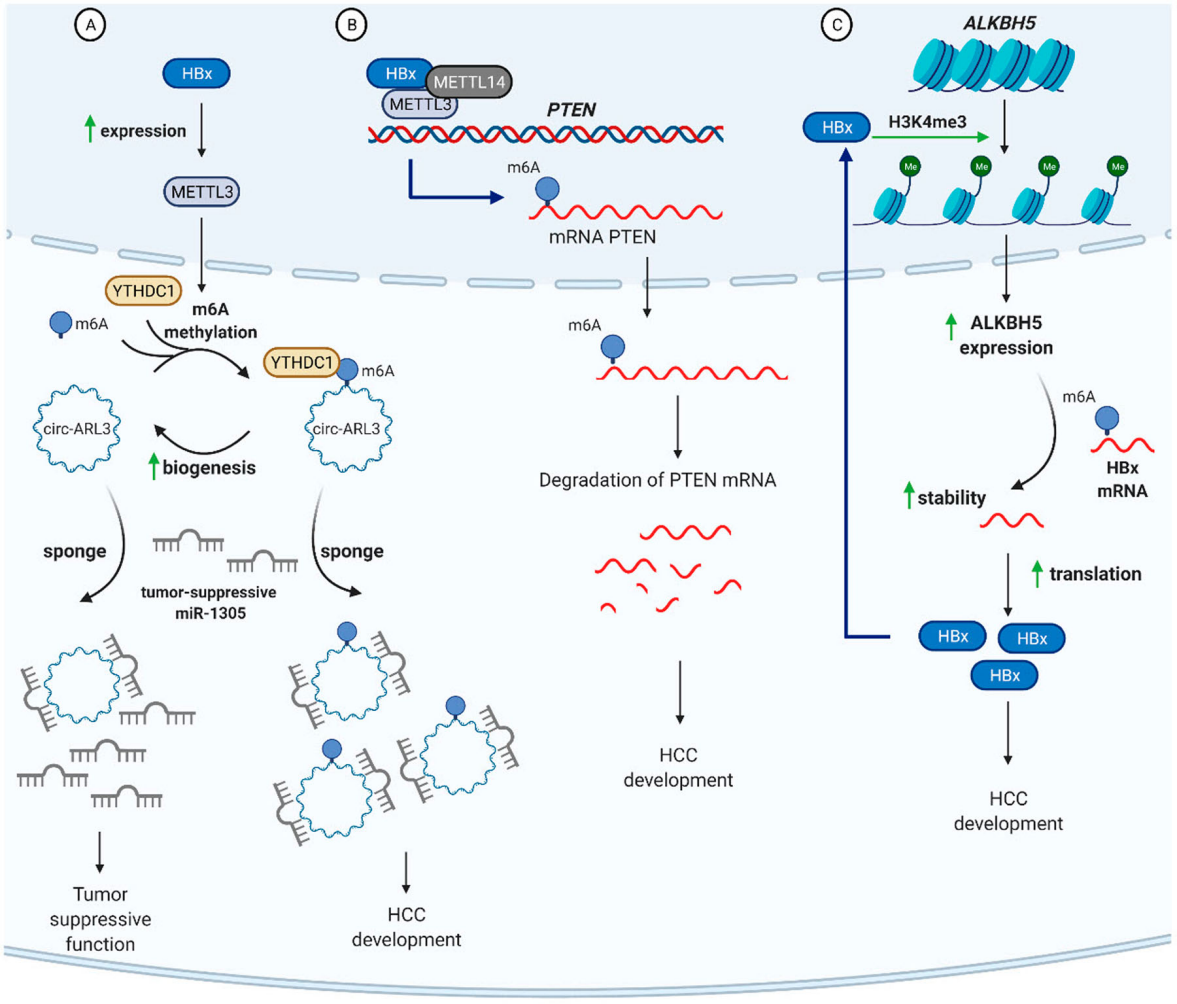
m6A-mediated mechanisms of HBV-induced liver carcinogenesis. (A) HBx upregulates METTL3 expression, resulting in m6A methylation of circ-ARL3 and its interaction with YTHDC1 reader protein. M6A modification enhances circ-ARL3 biogenesis and sponging of a strong tumour-suppressive miR-1305. Sponging inactivates miR-1305 and contributes to HCC development. (B) PTEN is a potent tumour suppressor. HBx-mediated m6A modification of PTEN mRNA results in PTEN mRNA degradation and loss of its function*.* (C) HBx stabilizes WD repeat-containing protein 5 (WDR5) protein, a subunit of histone H3 lysine 4 methyltransferase complex, promotes H3K4me3 histone modification of ALKBH5 promoter and upregulation ALKBH5 mRNA expression. ALKBH5 catalyses demethylation of HBx mRNA, increasing its stability and production of pro-oncogenic HBx protein. As such, HBx and ALKBH5 form a positive feedback loop and can be regarded a self-accelerating mechanism of HCC development.

A plethora of recent studies demonstrated that m6A modification plays a role in cancer by modifying oncogene and tumour suppressor RNA. Over 70% of HCC patients are HBV-infected [53]. The general concept is that HBV induces HCC directly (by increased genomic instability, insertional mutagenesis, pro-oncogenic effects, and triggering common mechanisms of cell transformation) and indirectly (via dysregulated immune responses and liver necro-inflammation) [54]. A new factor of HCC progression is m6A modification of cellular RNA [55]. A number of HBV-related RNA was found to be m6A modified and implicated in liver cancer [46,56]. Circular RNAs (circRNA) are non-coding RNA molecules that affect gene expression by competitively binding miRNA, thus changing miRNA efficacy in regulating mRNA levels [57]. This interactive circRNA-miRNA-mRNA network contributes to development and progression of many cancers. HBx increases m6A modification of circ-ARL3, a factor which binds tumour-suppressive miRNA-1305 and enhances the activity of oncogenes, resulting in the interaction of circ-ARL3 with YTHDC1 and increased biogenesis (Figure 4(A)) [56]. Increased levels of circ-ARL3 positively correlate with HCC malignancy and are associated with poor prognosis. HBx-induced dysregulation of the circ-ARL3/miRNA-1305 axis may thus be an important driver of HCC. M6A methylation of circ-ARL3 and possibly other malignancy-associated factors may be utilized as biomarkers for cancer prognosis and to guide the choice of therapeutic strategies [58].

Long non-coding RNA (lncRNA) is the type of RNA that is transcribed but is not translated into a protein. Instead of serving as a template for protein synthesis, lncRNAs appear to regulate gene expression and may contribute to cancer [59]. LncRNAs are frequently m6A-modified reducing lncRNAs stability and accelerating decay [59]. Recently, Zhu et al., also found that expression of TRP ion channel-related gene anti-sense RNA 1 (TRPC7-AS1) lncRNA was highly expressed and had low levels of m6A modification in HCC patients and hepatoma cell lines, indicating that m6A may play a role in the regulation of TRPC7-AS1 lncRNA expression [60]. TRPC7-AS1 lncRNA was thus proposed as a potential therapeutic or diagnostic marker. A plethora of lncRNAs is implicated in HBV replication and HCC development (e.g. lncRNA HOTAIR, UCA1, Linc00152, TUC338, MALAT1, and others) [61]. Whether these lncRNAs are regulated in an m6A manner is to be defined.

In a recent report, HBV-induced m6A modification of PTEN mRNA has been proposed as a new driver of HCC development (Figure 4(B)) [46]. Consistent PTEN protein levels are important in inhibiting carcinogenesis; indeed, PTEN-knockout mice develop HCC [62]. HBx protein, a recognized promoter of cancer, recruits METTL3/METTL14, and induces m6A methylation of PTEN mRNA, thereby destabilizing it and reducing PTEN protein expression. The resulting drop in PTEN protein production contributes to cancer development. Similar effect on PTEN mRNA stability was also observed in a model of HCV, another virus that causes HCC [46].

Fang et al. (2020) identified that aberrant expression of RBM15 and HNRNPA2B1 (mediators of m6A methylation) can be used for diagnosing and assessing the risk of metastasis in HBV-related HCC patients [63]. IGF2BP3 m6A reader recognizes m6A-modified HBV pgRNA, increasing its stability [64], while elevated pgRNA levels upregulate IGF2BP3 expression by a positive feedback loop. The resulting high levels of serum pgRNA are associated with poor prognosis and can be used to predict HCC recurrence in CHB patients. Overproduced ALKBH5 promotes tumour growth by forming a positive feedback loop with HBx protein: HBx triggers upregulation of ALKBH5 via H3K4me3 epigenetic modification of ALKBH5 promoter, causing the removal of m6A from HBx-encoding HBV RNA, leading to stabilization and intensive translation of HBx (Figure 4(C)). This self-promoting HBx/ALKBH5 loop may be used as an potential prognostic indicator and a novel therapeutic target for treating liver cancer.

Overall, aberrant expression of m6A factors has been reported in many different types of cancer, including HCC, which may be interesting both for prognostic purposes and for identifying novel molecular drug targets.

## Lessons learned from M6A in other viral infections

Factors of the m6A machinery have different (and often opposing) roles in viral replication. As outlined above, knocking down METTL3/METTL14 enhances the production of HBV proteins, whereas depleting FTO and ALKBH5 has the opposite effect. The same findings were observed during HIV-1 [65], HCV [66] and SARS-CoV-2 replication [32]. In contrast, depleting METTL14 suppresses Kaposi sarcoma-associated herpesvirus (KSHV) replication [67] and expression of Epstein–Barr virus transcripts [68].

A paramount role in the interplay between m6A and RNA activities is assigned to m6A readers. HBV transcripts are bound by YTHDF factors promoting their degradation and by YTHDC1, which facilitates nuclear export of m6A-marked viral RNA. YTHDF1-3 proteins can interact with m6A-modified HIV-1 RNA, suppressing or promoting viral replication [65]. YTHDF2 protein negatively regulates SARS-CoV-2 replication [32] and production of HCV particles [66] and, conversely, induces replication of simian virus 40 [69] and production of KSHV virions [67]. The common thought is that the effects of these factors are related to their intracellular localization, stoichiometry, presence of additional co-factors and, possibly, cell systems used.

M6A modification of mRNA plays an important role in regulating mRNA trafficking by increasing nuclear export of both viral and cellular RNAs. Indeed, during retrovirus infection, m6A modification of the RRE element increases its affinity for Rev protein and promotes nuclear export of HIV-1 RNA into the cytoplasm [65]. In contrast, m6A modification of HCV RNA was shown to retain m6A-modified HCV RNA at viral factories and attenuate viral replication [66].

Adding m6A modifications to RNA contributes to regulation of viral and cellular RNA splicing in viral infections by recruiting splicing machinery and regulating alternative splicing events [70]. Splicing is influenced by METTL3, WTAP, FTO, and ALKBH5, as well as the reader protein YTHDC1 [71]. As such, m6A regulates splicing and processing of adenovirus late transcripts [72] and splicing of ORF50 pre-mRNA of KSHV [73]. The role of m6A in splicing HBV transcripts is yet to be defined. It has been shown that HBV can generate numerous splice variants of its viral RNA, but their role in the HBV life cycle and pathogenesis of chronic HBV infection is scantly investigated.

Infection of primary human hepatocytes with HBV alters m6A-RNA methylation of many host factors, including PTEN, PTGFRN, POP4, RMB4B, etc. [46]. This indicates that either the host cell recognizes and tries to emanate the antiviral response (post-transcriptional antiviral response), or that HBV regulates gene expression post-transcriptionally to evade immunity. Indeed, SARS-CoV-2 infection affects the global RNA methylome in infected cells, in particular interferon-stimulated genes, although their expression level was mostly unaltered [32]. This indicates an important post-transcriptional level of regulation that influences virus-host interactions. The precise regulation of these interactions is still unclear for HBV infection and especially for co-infections with hepatitis D virus, HCV, HIV, and others [74].

## Future directions

M6A modification of viral and cellular transcripts is an important modulator of HBV replication and immunity, implicated in pathogenesis of CHB. Importantly, m6A modifications of HBV RNA could be one of the leading arms in the immune escape of the virus, contributing to viral persistence and chronicity [18]. HBx protein, the major factor contributing to HBV’s carcinogenic potential, exerts its pro-tumourigenic potential by adding m6A modifications to tumour suppressors (PTEN, circular RNAs, and miRNA function) [46,56]. The abundance of m6A machinery in HBV infection, as well as the abundance of m6A methylation in cellular transcripts, may serve as diagnostic predictors of HCC progression [63]. Although the evidence regarding the value of m6A-modified viral or cellular transcripts in predicting therapeutic outcomes for CHB is lacking, the connection between m6A, immune responses, and viral replication is clear. m6A modification of HBV RNA produced from viral integrations remain currently uncharacterized. Viral integrations are the sources of HBV proteins, mostly HBsAg and HBxAg, but also HBe/HBcAg (reviewed in [75]). HBxAg and HBsAg are important players modulating immune response and promoting liver carcinogenesis. It is intriguing to speculate that m6A modification of viral RNA transcribed from HBV DNA integrations may contribute to liver cancer and help to predict HCC occurrence/recurrence. Moreover, HBsAg is a clinically useful surrogate marker of viral replication. Loss of HBsAg indicates resolution of CHB or achievement of a functional cure. How m6A affects production of HBsAg from viral integrations and whether it could serve as a predictor for HBsAg loss is important to understand for the clinical management of CHB patients.

Another open question is the potential value of m6A modification in pgRNA packaging into viral capsids [13]. Interaction of viral Polymerase with 5′ serves as signal for pgRNA encapsidation. Upon interaction, pgRNA and viral Polymerase undergo conformational modifications [76]. If m6A alters pgRNA structure and affects its interaction with HBV Polymerase, requires further studies.

HBV pgRNA can be encapsidated and released in viral particles (serum pgRNA) [77]. Serum pgRNA can enter hepatocytes, but fails to establish infection [78], representing another dead end for the virus. It is unclear if serum pgRNA is m6A modified and whether re-infection of hepatocytes by serum pgRNA plays any role in HBV persistence and pathogenesis.

Analyzing the RNA methylome may be useful for solving the key clinical issues in the HBV field, such as establishing therapeutic outcomes for HBeAg-negative patients, predicting patient response to therapeutics [79], and assessing the risks of CHB progression. As novel molecular technologies are being rapidly introduced into clinical practice, modification of the m6A state of viral and cellular transcripts could prove useful for treating viral infections and immune dysfunctions.

An important point in the translation of m6A results into clinical practice is the robustness and accuracy of m6A detection methods. M6A modifications can be detected by RNA immunoprecipitation with specific anti-m6A antibodies followed by high-throughput sequencing by such methods as methylated RNA immunoprecipitation sequencing (MeRIP-seq)/m6A-seq. However, these methods require large amounts of input RNA and do not determine the location of specific m6A sites, allowing mapping of m6A sites only with a 100 nt resolution. Greater resolution can be achieved by UV crosslinking of anti-m6A antibody to RNA (miCLIP) [3] or m6A CLIP. miCLIP allows mapping of m6A residues with a single nucleotide resolution. miCLIP2 method coupled with machine learning analysis was optimized for low input samples. Single nucleotide positioning of m6A sites has also become possible with the use of third-generation sequencing technologies, such as Oxford Nanopore Technologies and PacBio. The former relies on identification of kinetic changes of reverse transcriptases encountering m6A-modified nucleotides, whereas the latter identifies m6A sites due to the differences in raw current intensities between m6A-modified and «blank» nucleotides. Enzyme-based methods, such as MATZER-seq and m6A-REF use MazF endoribonuclease that preferentially cuts ACA RNA motifs without m6A. As such, m6A sites can be detected by the reduction in enzyme cleavage efficiency. A fusion protein-based method, named deamination adjacent to RNA modification targets (DART-seq), takes advantage of the YTH protein fused to APOBEC1 deaminase. YTH domain recognizes m6A motifs, while APOBEC1 converts cytosine to uracil, detected by deep sequencing. Although a substantial progress has been achieved in m6A detection technologies, they all suffer from different limitations, such as the need to use high amounts of the starting materials, non-specific interaction of anti-m6A antibodies, complex library preparation, sequence-specific biases and others (reviewed in [80]). The need for intensive and highly specialized personnel, complex bioinformatic analysis and variable reproducibility compromise the utility of these methods for clinical practice. Further progress in m6A detection technologies and analysis is mandatory for clinical use.

Although discovered half a century ago, m6A modifications are hardly used in diagnostic and therapeutic approaches. Novel molecular tools and technologies have recently started drawing particular attention to this RNA epigenetic mark and its utility in the clinical setting. Detailed characterization of m6A alterations in HBV chronic infection and progression hold a lot of promise from clinical perspective.
